# Direct Comparison of Autologous and Allogeneic Transplantation of iPSC-Derived Neural Cells in the Brain of a Nonhuman Primate

**DOI:** 10.1016/j.stemcr.2013.08.007

**Published:** 2013-09-26

**Authors:** Asuka Morizane, Daisuke Doi, Tetsuhiro Kikuchi, Keisuke Okita, Akitsu Hotta, Toshiyuki Kawasaki, Takuya Hayashi, Hirotaka Onoe, Takashi Shiina, Shinya Yamanaka, Jun Takahashi

**Affiliations:** 1 Center for iPS Cell Research and Application (CiRA), Kyoto University, Kyoto 606-8507, Japan; 2 PRESTO, Japan Science and Technology Agency, Kawaguchi 332-0012, Japan; 3 Institute for Integrated Cell–Material Sciences (iCeMS), Kyoto University, Kyoto 606-8501, Japan; 4 Bio-function Imaging Team, RIKEN Center for Life Science Technologies (RIKEN CLST), Kobe 650-0047, Japan; 5 Functional Architecture Imaging Unit, RIKEN Center for Life Science Technologies (RIKEN CLST), Kobe 650-0047, Japan; 6 Department of Basic Medical Science and Molecular Medicine, Tokai University School of Medicine, Isehara 259-1143, Japan; 7 Department of Biological Repair, Institute for Frontier Medical Sciences, Kyoto University, Kyoto 606-8507, Japan; 8 Department of Neurosurgery, Kyoto University Graduate School of Medicine, Kyoto 606-8507, Japan

## Abstract

Induced pluripotent stem cells (iPSCs) provide the potential for autologous transplantation using cells derived from a patient’s own cells. However, the immunogenicity of iPSCs or their derivatives has been a matter of controversy, and up to now there has been no direct comparison of autologous and allogeneic transplantation in the brains of humans or nonhuman primates. Here, using nonhuman primates, we found that the autologous transplantation of iPSC-derived neurons elicited only a minimal immune response in the brain. In contrast, the allografts caused an acquired immune response with the activation of microglia (IBA-1^+^/MHC class II^+^) and the infiltration of leukocytes (CD45^+^/CD3^+^). Consequently, a higher number of dopaminergic neurons survived in the autografts. Our results suggest that the autologous transplantation of iPSC-derived neural cells is advantageous for minimizing the immune response in the brain compared with allogeneic grafts.

## Highlights

• iPSC-derived autografts cause only a minimal immune response in the primate brain
• Autografts have advantages over allografts even at an immune-privileged site
• Dopamine neurons survive even in allografts without immunosuppression

## Graphical Abstract

**Figure:**
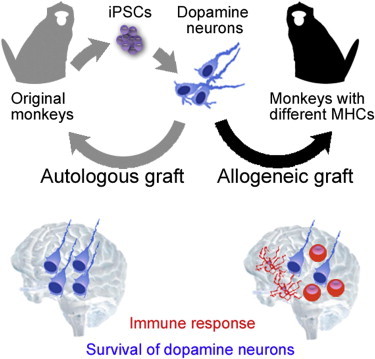

## Teaser

The autologous transplantation of iPSC-derived neurons elicited only a minimal immune response in the brain. In contrast, the allografts caused an acquired immune response with the activation of microglia (IBA-1^+^/MHC class II^+^) and the infiltration of leukocytes (CD45^+^/CD3^+^). Autologous transplantation is advantageous for minimizing the immune response compared with allogeneic grafts.

## Introduction

In recent studies, murine induced pluripotent stem cell (iPSC)-derived teratomas in the subcutaneous space induced an immune response in syngeneic mice (Zhao et al., 2011). In contrast, syngeneic transplantation of skin and bone marrow tissues (Araki et al., 2013) or endothelial, hepatic, and neuronal cells (Guha et al., 2013) derived from iPSCs showed a limited or no immune response, respectively. These rodent studies investigated the immunogenicity of teratomas, chimeric mouse-derived tissues, or ectopic grafts, but did not convincingly simulate the clinical situation.

Parkinson’s disease is one of the most promising targets for cell therapy with pluripotent stem cells, in which differentiated dopaminergic (DA) neurons are transplanted into the putamen of a patient’s brain (Lindvall and Björklund, 2011). In order to assess the immunogenicity of iPSC-derived neural cells in a primate brain, we generated iPSCs from four cynomolgus monkeys and directly compared the autologous and allogeneic transplantation of iPSC-derived neural cells.

## Results

### iPSCs Derived from Nonhuman Primates Differentiate into DA Neurons

**Figure 1:**
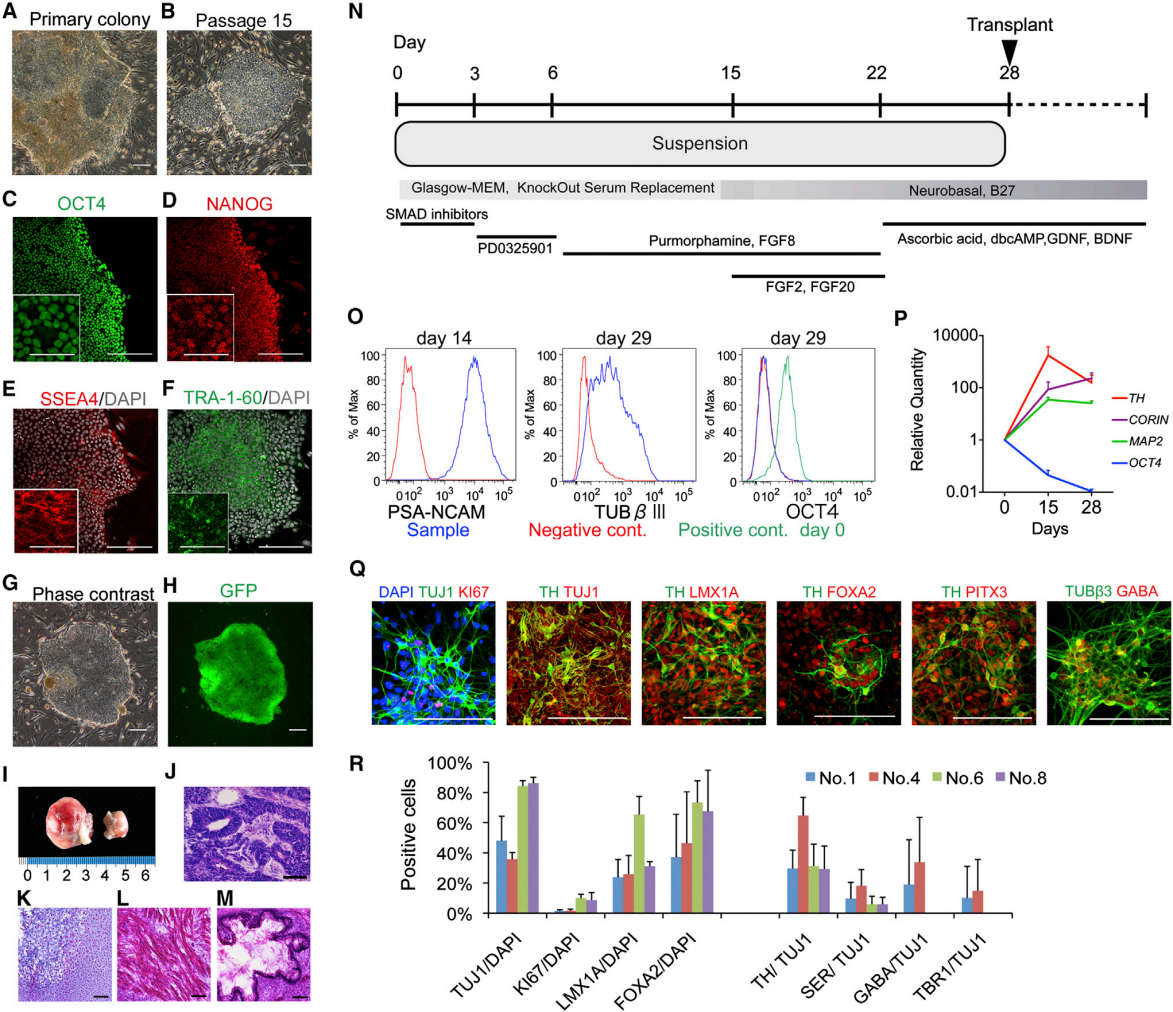
Characterization of Primate iPSCs and iPSC-Derived Neurons (A–H) Phase-contrast images (A, B, and G) and immunostaining for pluripotent markers (C–F) of iPSCs (T7). GFP was detected during live imaging (G and H) in the same field. (I–M) Teratoma formation at 3 months after transplantation in the testes of SCID mice. H&E staining of the sections showed histological features of the neuroepithelium (J), cartilage (K), muscle (L) and gut-like epithelium (M). (N) The protocol used for neural differentiation. (O) Expression analyses of neural markers and Oct4 by flow cytometry. The negative control was a cell sample stained only by secondary antibody (PSA-NCAM) or the isotype control (TUBβIII and OCT4). The positive control for OCT4 was undifferentiated (day 0) iPSCs. (P) qPCR for the differentiation of donor cells. The data are shown as the means ± SD (n = 4 independent experiments). (Q) Immunostaining of primate iPSC-derived neurons on day 39. (R) Quantification of immunocytochemical analyses for each iPSC line. Data are shown as the means ± SD (n = 3 independent experiments). SER, serotonin, TUBβIII, β-tubulin class III. Scale bars: 200 μm in (A)–(H), 50 μm in insets of (C)–(F), 100 μm in (J)–(M) and (Q). See also Figure S1 and Tables S2 and S3.

For the first two animals (Nos. 1 and 4), we established iPSCs from fibroblasts derived from the oral mucosa using retroviral vectors (Okita et al., 2011). For the other two animals (Nos. 6 and 8), we used peripheral blood mononuclear cells (PBMCs) with nonintegrating episomal vectors (Okita et al., 2013). We selected the best clone from each animal according to the following criteria: a stable embryonic stem cell (ESC)-like morphology of the colonies after passaging, expression of pluripotent markers, few or no integrated transgenes (Figures 1A–1F; Figure S1 available online), and the potential for stable neural differentiation. A PCR analysis revealed that all of the clones with retroviral vectors showed apparent expression of remaining transgenes (Figures S1C and S1D), whereas the clones with episomal vectors never did (Figure S1F). To detect the iPSC-derived cells in a brain, we introduced GFP (Figures 1G and 1H). The selected clones of iPSCs had the potential to generate teratomas in the testes of a severe combined immunodeficiency (SCID) mouse within 12 weeks (Figures 1I–1M).

To efficiently generate DA neurons from monkey iPSCs, we modified previously described protocols (Eiraku et al., 2008; Chambers et al., 2012; Morizane et al., 2011). Briefly, dissociated iPSCs were incubated in ultralow-attachment 96-well plates in medium containing inhibitors of bone morphogenetic protein (BMP) and Activin/NODAL signaling to initiate neural induction. To induce differentiation of the cells toward midbrain DA neurons, purmorphamine/FGF8 and FGF2/FGF20 were added sequentially (Figure 1N). During differentiation, the expression of a pluripotent marker (*OCT4*) gradually decreased, whereas the levels of neural and DA markers increased (Figures 1O and 1P). The differentiated neurons expressed markers characteristic of midbrain DA neurons (LMX1A, FOXA2, TH, and PITX3; Figures 1Q and 1R). Besides DA neurons, other types of neurons, such as serotonergic and GABAergic neurons, as well as proliferating neural progenitors positive for KI67, were also observed as minor populations (Figures 1Q and 1R). OCT4 was not detected by immunocytochemistry or flow cytometry in the differentiation culture even from the retroviral iPSCs (Figure 1O).

### Expression of Major Histocompatibility Complex by Monkey iPSCs In Vitro

**Figure 2:**
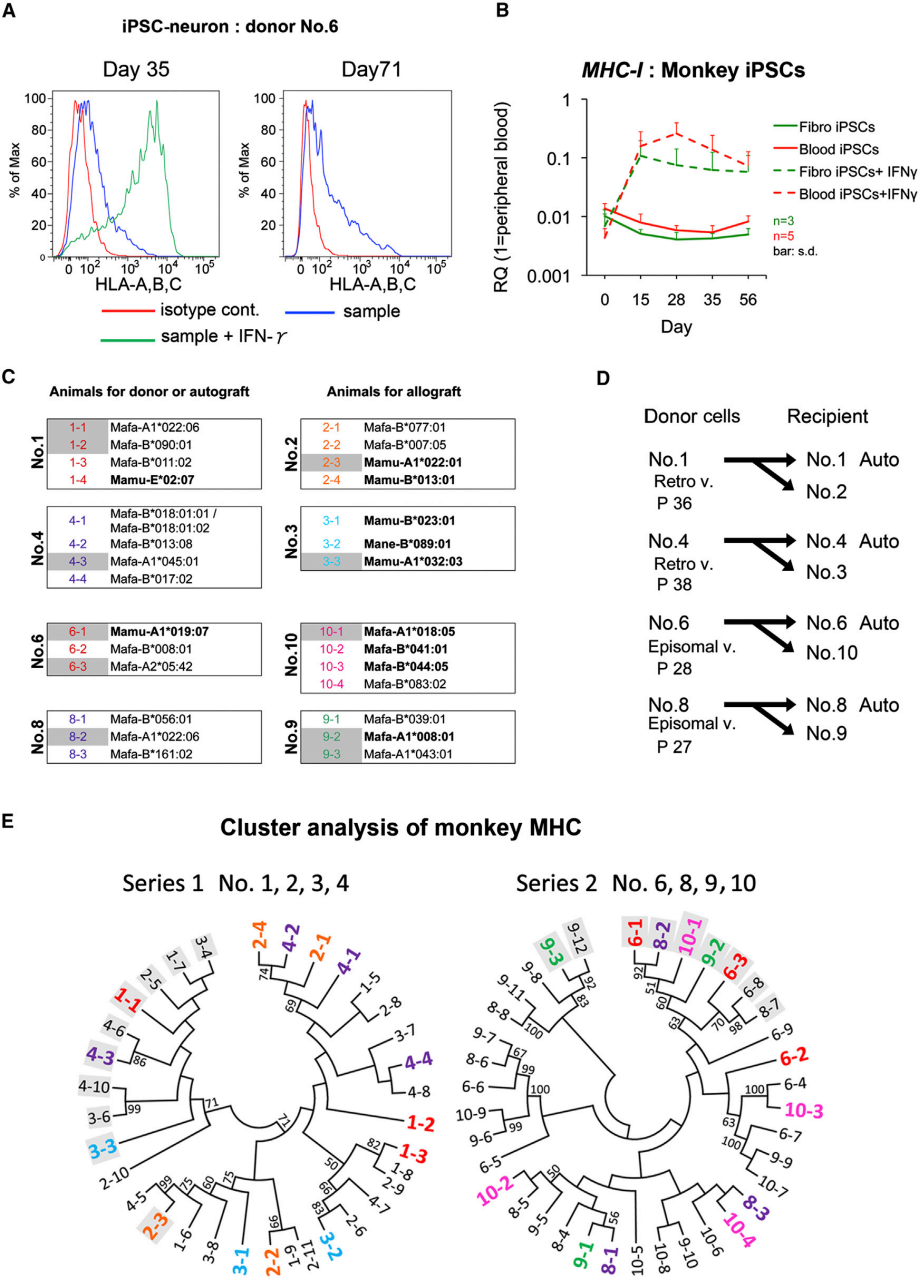
Identification of MHC Expression and Typing of Donor Cells (A) Flow-cytometric analyses for MHC-I (HLA-A, HLA-B, and HLA-C). Incubation of the cells with IFN-γ for 48 hr increased the MHC-I expression (green). (B) Temporal MHC-I expression analysis of monkey iPSCs by qPCR. The data were obtained from three (n = 3 for fibro iPSCs) or five (n = 5 for blood iPSCs) different experiments. Data are shown as the means ± SD. (C and E) Monkey MHC-I (Mafa) allele sequences detected by next-generation sequencing (C) and cluster analysis of the monkey MHCs (E). The colored letters indicate a comparatively high expression level of the MHC-I allele, comprising >10% of cDNA sequence reads. The gray background indicates the *MHC-A* allele, and the others indicate the *MHC-B* allele. (D) Combinations of donor cells and recipient animals. Episomal v., established with episomal vectors; P, passage number just before starting the differentiation of donor cells; Retro v., established with retrovirus vectors. See also Figure S4D and Tables S1–S3.

Next, we investigated the expression of major histocompatibility complex class I (MHC-I) in iPSC-derived neural cells. Flow cytometry using antibodies against human leukocyte antigen (HLA)-A, HLA-B, and HLA-C revealed that mature neurons on days 35 and 71 expressed only a low level of MHC-I, and that the expression was enhanced in response to interferon gamma (IFN-γ: 25 ng/ml for 48 hr; Figure 2A). The expression level of the mRNAs was approximately 1:100 compared with peripheral blood cells in both fibroblast- and blood-cell-derived iPSCs, which was again increased by exposure to IFN-γ (Figure 2B). These results suggest that donor-derived neurons could express MHC-I when INF-γ was secreted by the host brain under inflammatory conditions.

To ensure that the MHC-Is of the host animal and donor cells were mismatched in the allotransplantation cases, we performed genotyping of the expressed *MHCs* from the monkeys, which were purpose-bred, second-generation (F2), captive-born animals. As shown in Figures 2C and 2E, and Table S1, each monkey expressed different MHCs in terms of the A and B alleles. Based on these results, we chose the most mismatched combination for allotransplantation (Figures 2D and 2E).

### Autografts Elicit Only a Minimal Immune Response in the Primate Brain

We injected the iPSC-derived neural cells (day 28) into the original monkey as an autograft, and into the MHC-mismatched monkey as an allograft (Figure 2D). Each animal received six separate injections (∼8.0 × 10^5^ cells in a 4 μl suspension per tract) in the left striatum, and was observed for 3.5–4 months without immunosuppression. In the brain, both brain-resident microglia and circulating immune cells work as key players in immunological responses. Once the microglia are activated, they develop antigen-presenting activity. PK11195 selectively binds to the translocator protein that is expressed on activated microglia (Shah et al., 1994; Vowinckel et al., 1997). Therefore, positron emission tomography (PET) studies with [^11^C]PK11195 have been used to visualize brain inflammation in patients (Debruyne et al., 2003).

**Figure 3:**
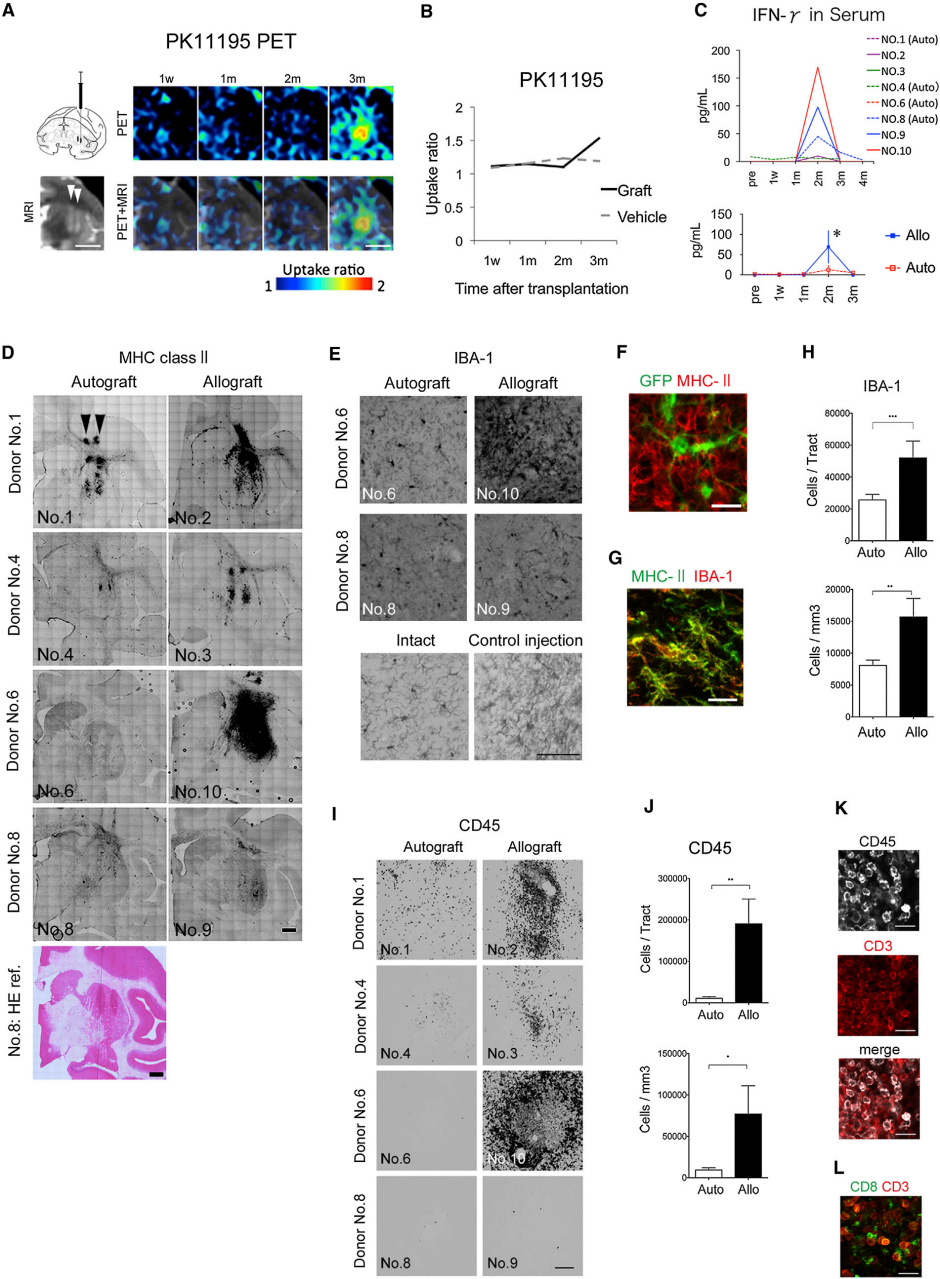
Immune Responses following Autologous or Allogeneic Transplantation (A and B) [^11^C]PK11195 PET study of the allografts in animal No. 10, in which the highest immune response was observed histologically. The illustration in (A) shows the method used for cell injection. (C) Temporal changes in the serum level of IFN-γ. Bottom: a two-way ANOVA was performed with Bonferroni’s multiple-comparisons test; n = 4 animals, ^∗^p < 0.05. Data are shown as the means ± SEM. (D–H) Histological analyses of the host-resident microglia. The image of H&E staining of animal No. 8 is shown as an anatomical reference for coronal sections (D). The arrowheads in (A) and (D) indicate the direction of the cell injections. (I–L) Histological analyses of infiltrating leukocytes. Scale bars: 1 cm (A), 2 mm (D), 100 μm (E and I), and 20 μm (F, G, K, and L). Quantitative data are presented as the means ± SEM (n = 22 tracts). Ratio paired t tests were performed for the auto- and allo-tracts. ^∗∗∗^p = 0.0003 (H, upper), ^∗∗^p = 0.0015 (H, lower), ^∗∗^p = 0.0035 (J, upper), ^∗^p = 0.0122 (J, lower). All of the PET, MRI, and histological images show coronal sections. See also Figures S2–S4 and Table S3.

In sequential PET studies, we observed increased uptake of [^11^C]PK11195 in one allograft (animal No. 10) at 3 months (Figures 3A and 3B). We could not detect any apparent uptake in the other animals or at any other time points (Figure S2). Intriguingly, the serum level of IFN-γ temporarily increased at 2 months after the transplant in three animals (Figure 3C). An immunofluorescence study conducted at 3.5–4 months showed that MHC-II^+^ cells were more frequently found in allografts than in autografts, especially in the monkey with increased uptake of [^11^C]PK11195 (Figure 3D, No. 10). The MHC-II staining never overlapped with that of GFP of the donor cells (Figure 3F), whereas it generally overlapped with that of IBA1 (Figure 3G), indicating that MHC-II was expressed by host-derived microglia. Consistently, the number and density of IBA1^+^ cells were higher in allografts than in autografts (Figures 3E, 3H, and S4C). An increase in the expression of MHC might trigger the recruitment of circulating immune cells, including T cells. An immunofluorescence study revealed that more CD45^+^ cells (a marker for pan-leukocytes) accumulated in allografts compared with autografts (Figures 3I and 3J). Most of the CD45^+^ cells were CD3^+^ T cells, and 60% of them were CD8^+^ killer T cells (Figures 3K and 3L). These findings suggest that an acquired immune response was elicited only in the allografts in the primate brain.

### DA Neurons Survive in Both Types of Grafts, but a Higher Number Are Observed in Autografts

**Figure 4:**
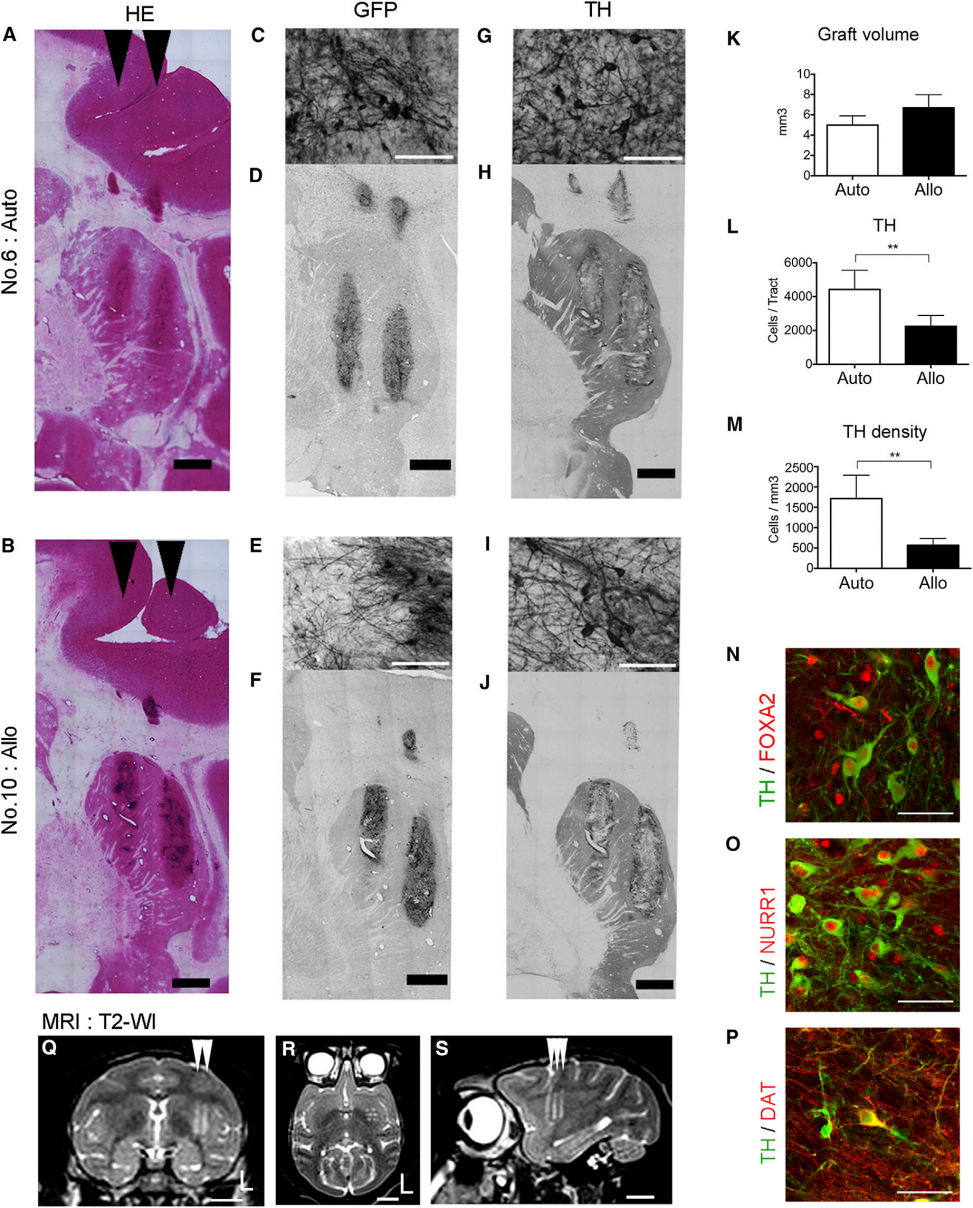
Graft Survival in a Primate Brain (A–P) Histological analyses of a brain section with H&E staining, and the immunohistochemical findings. (K) Quantitative analyses of the graft volume. (L and M) Survival of TH^+^ neurons in the grafts. (N and O) The TH^+^ neurons expressed markers of the midbrain phenotype: Foxa2 (N) and Nurr1 (O). (P) The DAT was also positively stained. (Q–S) Magnetic resonance images of a representative animal (No. 6, autograft) at 3 months after the transplant. The arrowheads indicate the directions of the cell injections. (Q) coronal, (R) axial, and (S) sagittal. The letter L indicates the left side. Scale bars: 2 mm (A, B, D, F, H, and J), 50 μm (C, E, G, I, N, O, and P), and 1 cm (Q–S). Quantitative data are presented as the mean ± SEM (n = 22 tracts). Ratio paired t tests were performed for the auto- and allo-tracts: ^∗∗^p = 0.0021 (L), ^∗∗^p = 0.0088 (M). See also Figure S4 and Table S3.

In order to evaluate the survival of the grafted cells, we performed MRI scanning and a histological analysis at 3–4 months posttransplantation (Figure 4). Hematoxylin and eosin (H&E) staining and immunostaining for GFP of the brain slices demonstrated that the grafted cells survived in both auto- and allotransplantation without immunosuppression (Figures 4A–4F). Furthermore, there were no significant differences in volume between the auto- and allografts (Figure 4K). Immunostaining for tyrosine hydroxylase (TH), a marker for DA neurons, revealed that a large number of DA neurons (4,428 ± 1,130 per tract, n = 22) survived in the autografts (Figures 4G, 4H, 4L, and 4M). The surviving DA neurons coexpressed the markers of a mesencephalic phenotype, such as FOXA2, NURR1, and the dopamine transporter (DAT) (Figures 4N–4P). Even in allografts without immunosuppression, the TH^+^ neurons survived well (2,247 ± 641 per tract, n = 22), but the number and density were lower than in autografts (Figures 4I, 4J, 4L, and 4M). We also found a small number of astrocytes (GFP^+^/GFAP^+^), as well as mature neurons (GFP^+^/NEUN^+^), in vivo (Figure S4B).

## Discussion

In this study, we induced DA neurons from monkey iPSCs by directed differentiation in vitro, and demonstrated that autologous transplantation of the iPSC-derived cells elicited only a minimal immune response in the nonhuman primate brain. Previous reports have suggested that either autologous grafts of iPSC-derived neural cells (Emborg et al., 2013; Maria et al., 2013) or allogeneic grafts of fetal ventral mesencephalic cells (Redmond et al., 2008) can survive in a primate brain without immunosuppression. None of these studies, however, directly compared the immunogenicity of autologous grafts with that of allogeneic ones. Our results clearly show differences in both immunogenicity and cell survival between these two types of grafts, and support the idea that immunosuppression is not necessary for autologous transplantation of iPSC-derived neural cells into the brain. The [^11^C]PK11195 PET study was useful for real-time visualization of this phenomenon. Furthermore, this technique can also be applied to patients in the clinical setting, which would help to determine when and if immunosuppressive drugs can be withdrawn.

Although we did not examine acute immune responses or inflammation within 48 hr, it is noteworthy that the responses were observed 2 or 3 months posttransplantation. In the case of iPSC-based transplantation, there are four possible mechanisms that can cause inflammatory and immune responses: (1) direct allorecognition of mismatched MHC or minor antigens of the donor cells, (2) indirect allorecognition through host-derived antigen-presenting cells, (3) expression of fetal antigens due to immature stem cells or remaining transgenes, and (4) mechanical damage rather than MHC mismatch. Because of the low expression level of MHC-I by the donor cells, direct allorecognition is unlikely to be the main cause. However, donor-cell-derived astrocytes (Figure S4B), which can express both MHC-I and MHC-II to recruit T cells in response to IFN-γ (Akesson et al., 2009; Chastain et al., 2011), could be observed in the grafts. Furthermore, it takes a longer time for astrocytes to differentiate than neurons. Therefore, although there was no apparent expression of either MHC-I or MHC-II by the grafted GFP^+^ cells, donor-derived astrocytes may have contributed to the direct reaction in the late stage. Considering the high expression level of MHC-II by host-derived microglia in the allografts (Figures 3D–3G), indirect allorecognition seems to have played a major role in the present study. This requires the internalization and processing of the alloantigens, which are then recognized in peptidic form bound to recipient MHC-II molecules, possibly accounting for the late onset of the immune response.

Autografts derived from iPSCs generated by retroviral vectors resulted in the accumulation of larger numbers of IBA1^+^ and CD45^+^ cells compared with those generated using episomal vectors, probably due to the residual expression of the transgenes (Figure S3). This indicates that residual transgenes can be immunogenic, and that it is therefore critical to use integration-free iPSCs. Mechanical damage caused by needle trauma can also activate host astrocytes and microglia to secrete proinflammatory cytokines, which recruit leukocytes. Consistently, we found IBA1^+^ cells along the needle tract in the animals that received control injections (Figures 3E and S4C), but this was limited to a small area and not likely to play a major role.

Another important finding is that, in spite of the immune responses mounted by the host brain, a substantial number of TH^+^ cells survived in the allografts. This is consistent with previous clinical reports of human fetal cell transplantation. Postmortem analyses of the patients revealed robust survival of DA neurons in spite of the fact that numerous immune cells were present around the graft (Kordower et al., 1997). In two double-blind clinical trials, immunosuppressive drugs were never used (Freed et al., 2001) or were withdrawn after 6 months (Olanow et al., 2003). In these cases, the cells from multiple fetuses were used without HLA matching, but more than 50,000 TH^+^ cells had survived after several years. Our quantitative PCR (qPCR) study in vitro showed that the expression of MHC-I increased in response to IFN-γ, but the expression level was still 1/10 that of untreated monkey peripheral blood cells (Figure 2B). The in vivo studies revealed that the serum level of IFN-γ increased at 2 months, and CD45^+^ cells (including CD8^+^ cells) accumulated in the allografts 3.5–4 months after the transplant. On the other hand, the levels of INF-γ in the cerebrospinal fluid (CSF) and the levels of tumor necrosis factor α (TNF-α) in both the serum and CSF were below the limit of detection by ELISA (data not shown). An immunofluorescence study did not reveal any apparent expression of MHC-I by the grafted cells (Figure S4A). Therefore, it is possible that the immune response in the primate brain was not strong enough to reject all of the donor cells. These findings closely correlate with the results of previous murine experiments (Hudson et al., 1994; Shinoda et al., 1995). To apply our findings to a more clinically relevant setting, we investigated the expression of HLA-I during neural differentiation of human ESCs (hESCs) and iPSCs by qPCR (Figure S4D). The expression level was 1/100 compared with that of human peripheral blood cells in both hESCs and iPSCs, and it was similarly elevated in response to IFN-γ. It is difficult to precisely compare immunogenicity in monkeys with that in humans, but the low expression level of MHC-I by the donor cells may account for the mild rejection in both monkey and human neural transplantation.

Our results indicate that autologous transplantation is beneficial in terms of the immune response and cell survival. However, this strategy is associated with higher costs and labor. An alternative method is allogeneic transplantation using HLA-matched iPSC stocks (Okita et al., 2011; Nakatsuji et al., 2008; Deleidi et al., 2011). Therefore, as a next step, it is critical to determine whether autografts have advantages over HLA-matched allografts and HLA-mismatched allografts with immunosuppression. To answer this question, it will be necessary to establish iPSCs from MHC-homozygous monkeys and transplant the iPSC-derived cells into monkeys with the identical MHC haplotype. The precise influence of HLA mismatch therefore needs to be explored in such future studies.

## Experimental Procedures

### Nonhuman Primates

Eight purpose-bred male cynomolgus monkeys (*Macaca fascicularis*) were used for iPSC generation and transplantation. The animal experiments were performed in accordance with the Guidelines for Animal Experiments of Kyoto University, the Institutional Animal Care and Use Committee of Kobe Institute in RIKEN, and the Guide for the Care and Use of Laboratory Animals of the Institute of Laboratory Animal Resources (Washington, DC, USA). See also Supplemental Experimental Procedures.

### Generation and Neural Differentiation of iPSCs

Fibroblasts from the oral mucosa were transfected retrovirally with five transgenes (*OCT3/4*, *SOX2*, *KLF4*, *L-MYC*, and *LIN28*) (Okita et al., 2011). PBMCs were transfected with a combination of plasmid vectors (*OCT3/4*, *SOX2*, *KLF4*, *L-MYC*, *LIN28*, shRNA for TP53, and *EBNA1*) as described previously (Okita et al., 2013). The primate iPSCs were maintained on mouse embryonic fibroblast feeders treated with mitomycin-C (Sigma-Aldrich). They were differentiated into DA neurons through the SFEBq method (Eiraku et al., 2008) with dual SMAD inhibitors (Chambers et al., 2012; Morizane et al., 2011; Figure 1N). The cells were transplanted on day 28 of differentiation. For in vitro analysis, the cells were dissociated with Accumax (Innovative Cell Technologies) on day 28 and cultured on eight-well glass chamber slides coated with poly-l-ornithine and laminin for an additional 11 days (for a total of 39 days).

### Genotyping of MHC

*MHC* genotypes were assigned by comparing the sequences with known *MHC* allele sequences released from the Immuno Polymorphism Database (http://www.ebi.ac.uk/ipd/index.html). See also Supplemental Experimental Procedures.

### Immunostaining and Histological Analyses

For in vivo studies, the fixed frozen brains were sliced at 40 μm thickness and immunologically stained via the free-floating method. The primary antibodies used are listed in Table S3. See also Supplemental Experimental Procedures.

### MRI and PET Studies

PET scans with [^11^C]PK11195 were performed with the use of an animal PET scanner (microPET Focus220; Siemens Medical Solutions) to identify the activation of microglia. High-resolution T1-weighted and T2-weighted images were obtained using a 3T MRI scanner (MAGNETOM Verio; Siemens AG) to identify the injection site of grafts in the postero-dorsal striatum and to evaluate graft survival. See also Supplemental Experimental Procedures.

### Transplantation

Floating aggregates (day 28) were harvested and dissociated into small clumps of 20–30 cells with Accumax. The cells were suspended in the last culture medium (Figure 1N), which was neurobasal medium with B27 with added ascorbic acid, dibutyryl-cAMP, glial-cell-line-derived neurotrophic factor, and brain-derived neurotrophic factor. We also added a ROCK inhibitor, Y27632, to increase the survival of the donor cells. The suspension was prepared at a concentration of 2 × 10^5^ cells/μl, and 4 μl of the suspension was injected through a 22-gauge needle with a Hamilton syringe. We made six (two coronal × three sagittal) tracts of injection in one side of the putamen. In total, 4.8 × 10^6^ cells per animal (8.0 × 10^5^ cells/tract × 6 tracts) were injected into one side of the putamen according to the coordinate decided by the MRI image of each monkey. The same volume of the culture medium was injected to the contralateral side as control. No immunosuppressant was used. Under deep anesthesia, the animals were sacrificed and perfused transcardially with 4% paraformaldehyde after 3.5–4 months of observation.

### Statistics

The data were expressed as the mean ± SD or mean ± SEM, and differences were tested by commercially available software Prism 6 (GraphPad). With regard to the histological data for the auto- and allo-tracts, after confirming normal distribution, we proceeded to perform statistical analyses with ratio paired t tests. Some tracts were omitted from the assessment because of technical problems; p values < 0.05 were considered to be significant.

## Supplemental Information

Document S1. Supplemental Experimental Procedures and Figures S1–S4 and Tables S1–S3
