# Supplementary material for: Direct Comparison of Autologous and Allogeneic Transplantation of iPSC-Derived Neural Cells in the Brain of a Nonhuman Primate
Source: Stem Cell Reports. 2013 Sep 26;1(4):283–92. doi: 10.1016/j.stemcr.2013.08.007 (PMC3849265; doi:10.1016/j.stemcr.2013.08.007)
Supplement: Document S1. Supplemental Experimental Procedures and Figures S1–S4 and Tables S1–S3 [file mmc1.pdf]

Stem Cell Reports, Volume *1*

Supplemental Information

Direct Comparison of Autologous and Allogeneic Transplantation of iPSC-Derived Neural Cells in the Brain of a Nonhuman Primate

Asuka Morizane, Daisuke Doi, Tetsuhiro Kikuchi, Keisuke Okita, Akitsu Hotta, Toshiyuki Kawasaki, Takuya Hayashi, Hirotaka Onoe, Takashi Shiina, Shinya Yamanaka, and Jun Takahashi

Inventory of supplemental information

Supplemental experimental procedures
Figure S1, Characterization of iPSCs, related to Figure 1
Figure S2, PK11195 PET study, related to Figure 3B
Figure S3, Quantitative analyses of IBA1+ and CD45+ cells, related to Figure 3
Figure S4, Histological analyses and HLA-1 expression of human pluripotent stem cells, related to Figures 2-4
Table S1, The cynomolgus macaque MHC class I allele sequences, related to Figure 2
Table S2, List of primers for PCR, related to Figures 1-2, S1, S4
Table S3, List of primary antibodies, related to Figures 1-4, S1, S4

SUPPLEMENTAL EXPERIMENTAL PROCEDURES

Non-human primate. Eight purpose-bred male cynomolgus monkeys (Macaca fascicularis) were used for iPSC generation and transplantation. Experiments using non-human primates always have a limitation of the animal number because of the issues of cost and ethics. In our experiments, we used eight primates in total and analyzed 24 grafts (six tracts/animal) for each type of the graft, but this sample number and the diversity of MHC types might not have been large enough to draw definitive conclusions.

Genotyping of MHC. Total RNA was isolated from the peripheral white blood cells using the TRIzol reagent (Invitrogen). cDNA was synthesized using the ReverTra Ace (TOYOBO). The MHC class I gene primer pairs used for human and macaque genes (Table S2) were designed based on generic sequences in exons 2 to 4 that amplify all known class I genes for RT-PCR amplification. After RT-PCR amplification using high fidelity KOD FX polymerase (TOYOBO), pyrosequencing of the PCR products was

carried out using the GS Junior system and amplicon sequencing protocol (Roche). *MHC* genotypes were assigned by comparing the sequences to known *MHC* allele sequences released from the Immuno Polymorphism Database (http://www.ebi.ac.uk/ipd/index.html).

Teratoma formation. Injection of iPSCs (1x10^6 cells per injection) into the testes of SCID mice generated teratoma. Three months after injection, the tumors were surgically dissected and fixed with 4% Paraformaldehyde (PFA). The tumor were sectioned with a cryostat and stained with hematoxylin/eosin (HE).

GFP transfection. The iPSCs were transfected GFP as a marker of the donor cells. *PB-EF1α-EGFP-IRES-PUROMYCIN* plasmid vector was transfected with FuGENE HD system. Puromycin was added to the medium for selection of transfected cells.

RT-PCR, qPCR and genomic PCR. Total RNA fraction was extracted using an RNeasy Mini kit (QIAGEN), and reverse-transcribed using Super ScriptⅢ First-Strand

Synthesis System (Invitrogen). For PCR amplification, reactions were performed using HotStarTaq (QIAGEN). Quantitative PCR reactions were carried out with Power Syber (Applied Biosystems) according to the manufacturer's instructions. The expression levels of each gene were normalized to that of *GAPDH* using the ΔCT method. Genomic DNA was extracted using the DNeasy Blood and Tissue Kit (QIAGEN). Primers used for the reactions are shown in Table S2.

Flowcytometery. The cells were stained with antibodies for cell surface marker. Dead cells and debris were excluded by 7-AAD (BD Biosciences) staining. For the antibodies against the fixed cells, we used Live/Dead Fixable Red Dead Cell stain kit (Invitrogen) and BD Perm/Wash buffer™ (BD Biosciences) according to the manufacture's protocol. The analysis was performed with BD LSRFortessa flow cytometer, FACSDiva software program (both from BD Biosciences) and FlowJo flow cytometry analysis software (Tree Star).

Immunostaining and hisotological analyses. For i*n vivo* studies, the fixed frozen brain

was sliced at 40μm thickness. They were immunologically stained by free-floating method. Primary antibodies used were listed in Table S3. For staining with CD3 antibody, we took an additional step for antigen retrieval in 10mM Citrate buffer at 100°C 40 min. Fluorescent staining with Alexa secondary antibodies (Invitrogen) or 3,3'-Diaminobenzidine (DAB) staining with biotinylated secondary antibody, and ABC Elite Kit (Vector Labs) was performed. The cells were visualized using a confocal laser microscope (Fluoview FV1000D, Olympus) for the fluorescence. The TissueFAXS Cell Analysis system (TissueGnostics) with a x5 objective lens was used to acquire tiled pictures for low magnification of the DAB and HE staining covering the whole hemisphere. A x20 objective lens was also used to acquire tiled pictures for higher magnification of the TH, IBA-1 and CD45 staining covering the whole grafts. To measure the graft volumes, low magnified tiled HE images were imported into the Image J software program (http://rsbweb.nih.gov/ij/) and the graft areas were quantified every 18 sections throughout the graft. The estimated graft volumes were calculated based on the thickness of the brain slices. The number of immunoreactive cells was quantified in every 18 section throughout the graft and surrounding tissue and corrected

by using the Abercrombie method.

MRI and PET study. PET scans with [^{11}C]PK11195 were performed by an animal PET scanner (microPET Focus220; Siemens Medical Solutions) to identify the activation of microglia. A high-resolution T1-weighted and T2-weighted images were obtained using a 3T MRI scanner (MAGNETOM Verio, Siemens AG) to identify the injection site of grafts in postero-dorsal striatum and to evaluate the graft survival. Although high signals in T2-weighted images are generally based on various tissue changes, such as edema, inflammation, etc., the grafted areas with the T2-high signals were very homogenous and clearly demarcated from the surrounding brain tissues, unlike inflammatory/edematous changes. In fact, the volumes of T2-high signal areas reflected those of the engrafted tissues, at least in our system, as shown in our comparative histological studies (Doi et al., 2012; Kikuchi et al., 2013).

ELISA. Serum and cerebrospinal fluid (CSF) were collected from animals. The concentration of interferon gamma (IFN-γ), and tumor necrosis factor alpha (TNF-α)

was measured with ELISA kit Monkey for each factor according to the manufacture's protocol (Invitrogen).

REFERENCES

Doi, D., Morizane, A., Kikuchi, T., Onoe, H., Hayashi, T., Kawasaki, T., Motono, M., Sasai, Y., Saiki, H., et al. (2012). Prolonged Maturation Culture Favors a Reduction in the Tumorigenicity and the Dopaminergic Function of Human ESC-Derived Neural Cells in a Primate Model of Parkinson's Disease. Stem Cells *30*, 935-945.

Kikuchi, T., Morizane, A., Doi, D., Onoe, H., Hayashi, T., Kawasaki, T., Saiki, H., Miyamoto, S., and Takahashi, J. (2011). Survival of Human Induced Pluripotent Stem Cell--Derived Midbrain Dopaminergic Neurons in the Brain of a Primate Model of Parkinson's Disease. J Parkinson's Disease *1*, 395-412.

Figure S1

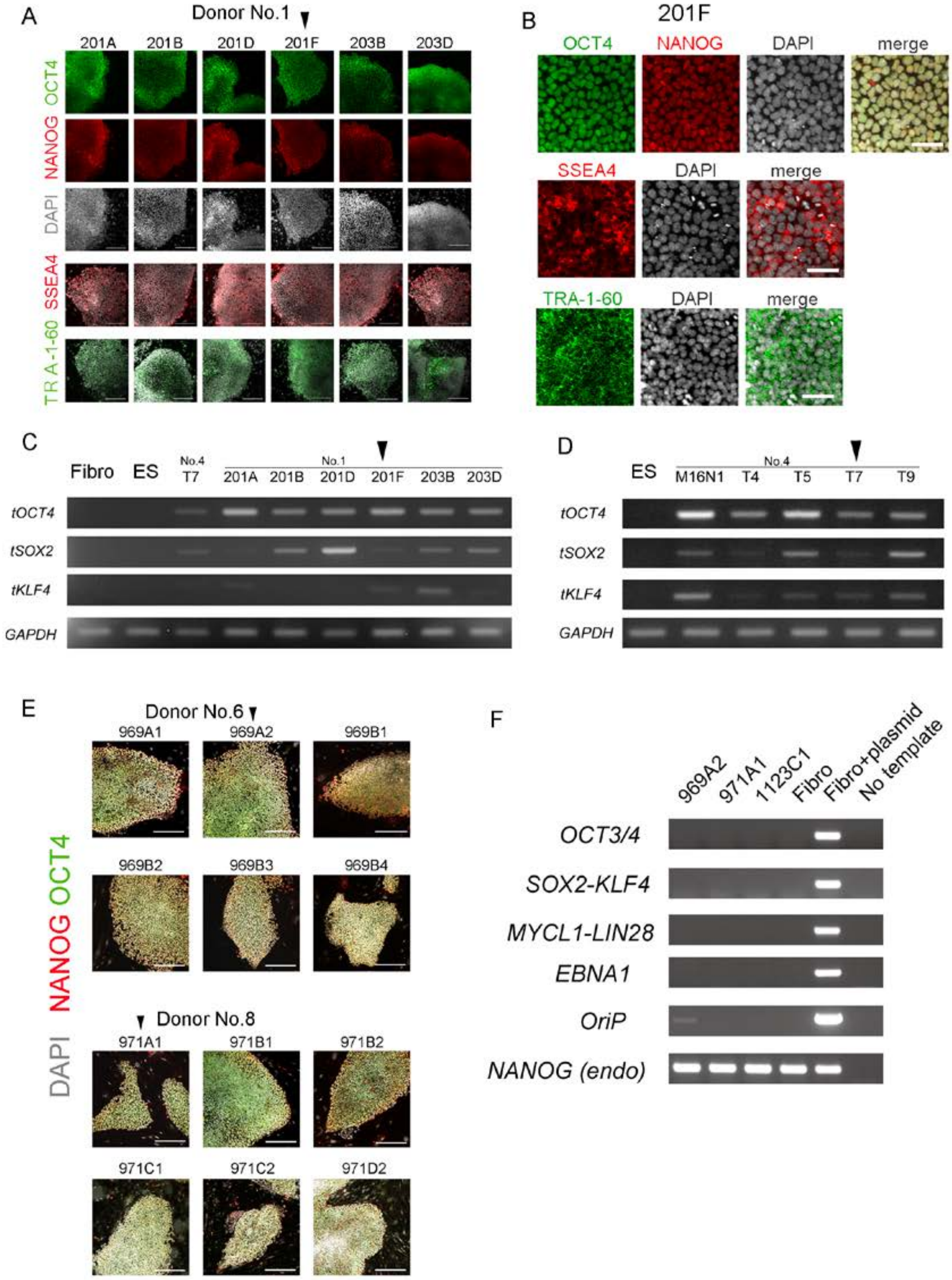

A
Donor No.1
201A
201B
201D
201F
203B
203D
OCT4
NANOG
DAPI
SSEA4
TRA-1-60
B
201F
OCT4
NANOG
DAPI
merge
SSEA4
DAPI
merge
TRA-1-60
DAPI
merge
C
Fibro
ES
No.4
T7
No.1
201A
201B
201D
201F
203B
203D
tOCT4
tSOX2
tKLF4
GAPDH
D
ES
No.4
M16N1
T4
T5
T7
T9
tOCT4
tSOX2
tKLF4
GAPDH
E
Donor No.6
969A1
969A2
969B1
969B2
969B3
969B4
Donor No.8
971A1
971B1
971B2
971C1
971C2
971D2
DAPI NANOG OCT4
F
969A2
971A1
1123C1
Fibro
Fibro+plasmid
No template
OCT3/4
SOX2-KLF4
MYCL1-LIN28
EBNA1
OriP
NANOG (endo)

Figure S2

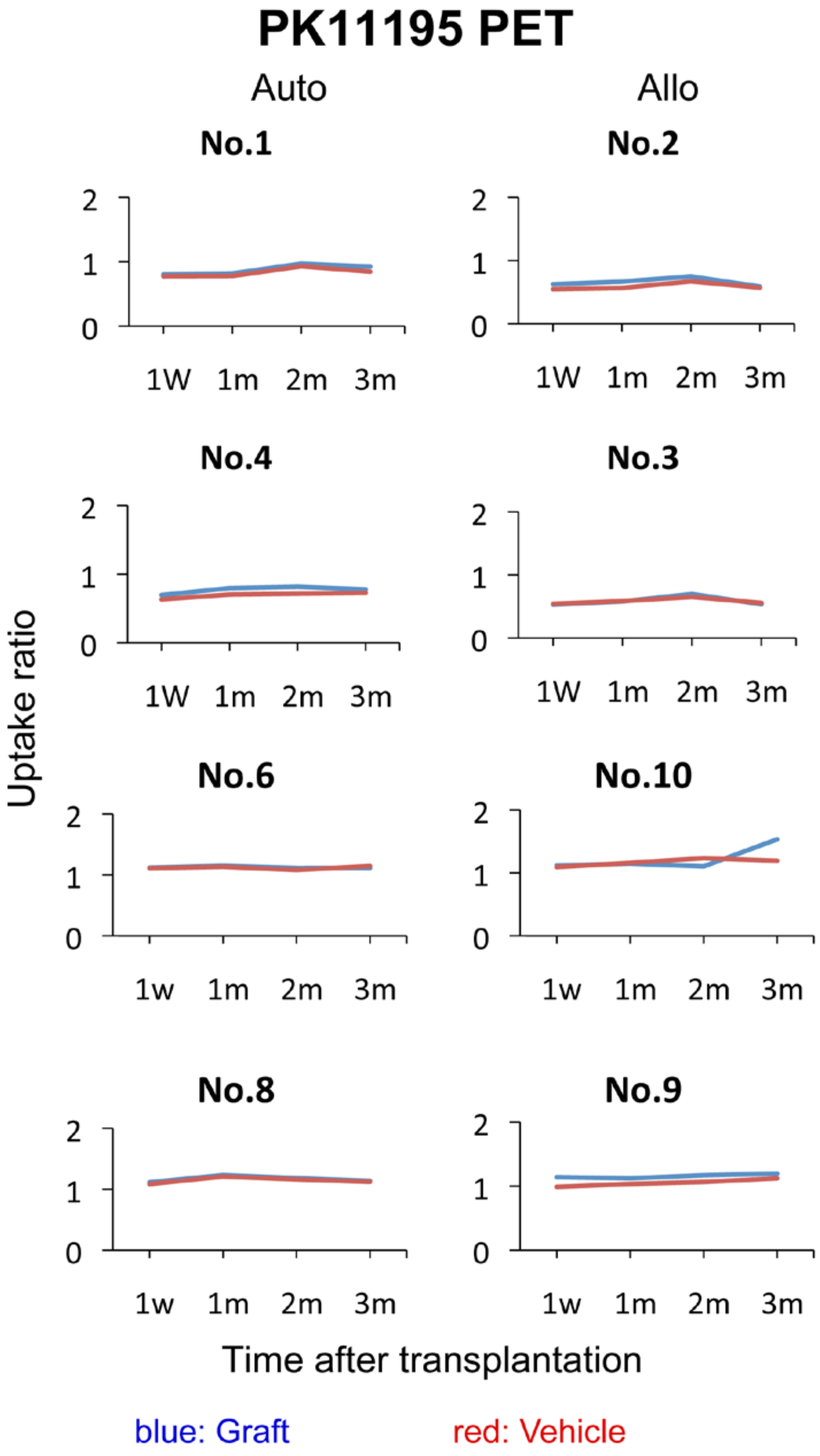
PK11195 PET
Auto
Allo
No.1
No.2
No.4
No.3
No.6
No.10
No.8
No.9
Uptake ratio
0
1
2
1W
1m
2m
3m
1w
Time after transplantation
blue: Graft
red: Vehicle

Figure S3

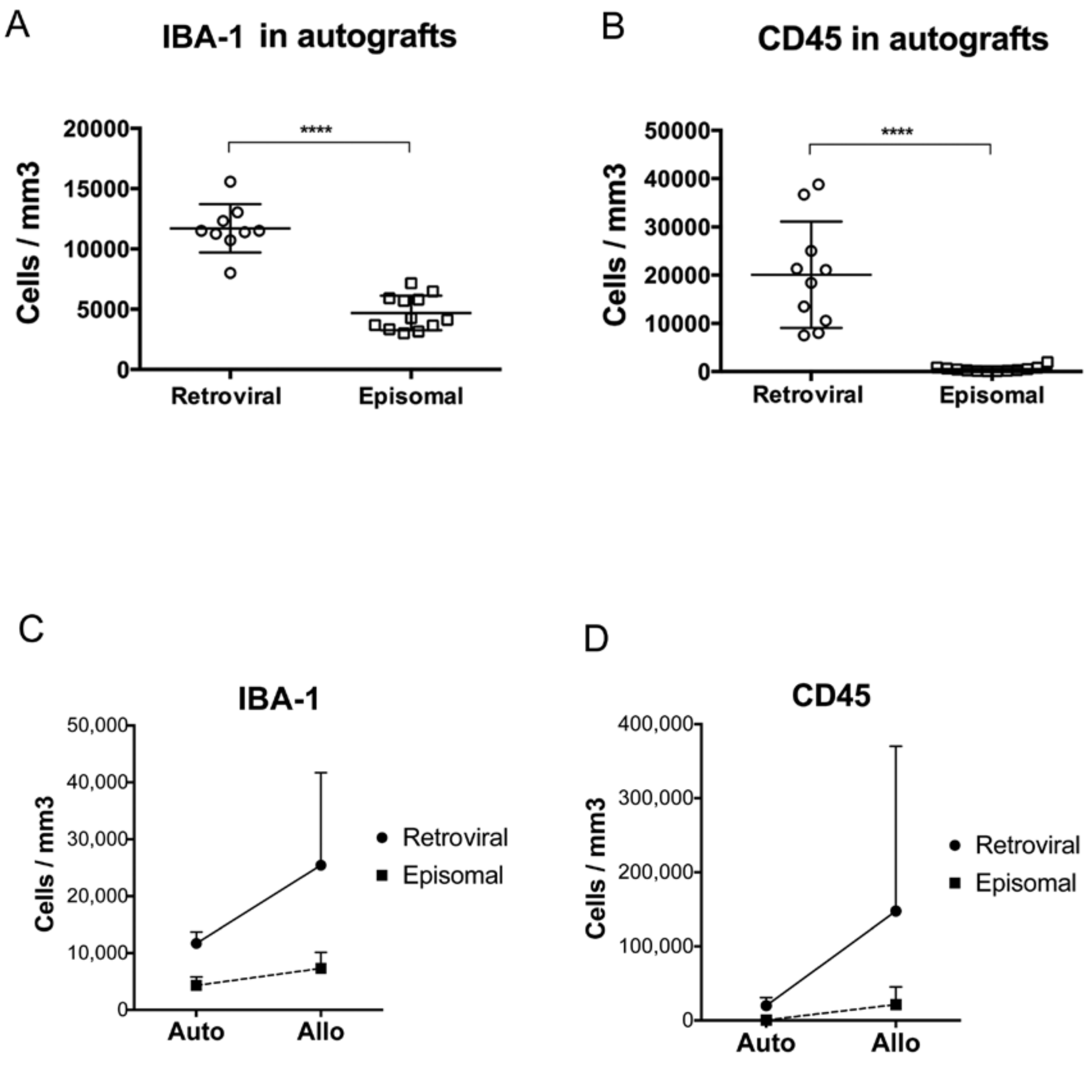
A
IBA-1 in autografts
Cells / mm3
20000
15000
10000
5000
0
****
Retroviral
Episomal
B
CD45 in autografts
Cells / mm3
50000
40000
30000
20000
10000
0
****
Retroviral
Episomal
C
IBA-1
Cells / mm3
50,000
40,000
30,000
20,000
10,000
0
Auto
Allo
Retroviral
Episomal
D
CD45
Cells / mm3
400,000
300,000
200,000
100,000
0
Auto
Allo
Retroviral
Episomal

Figure S4

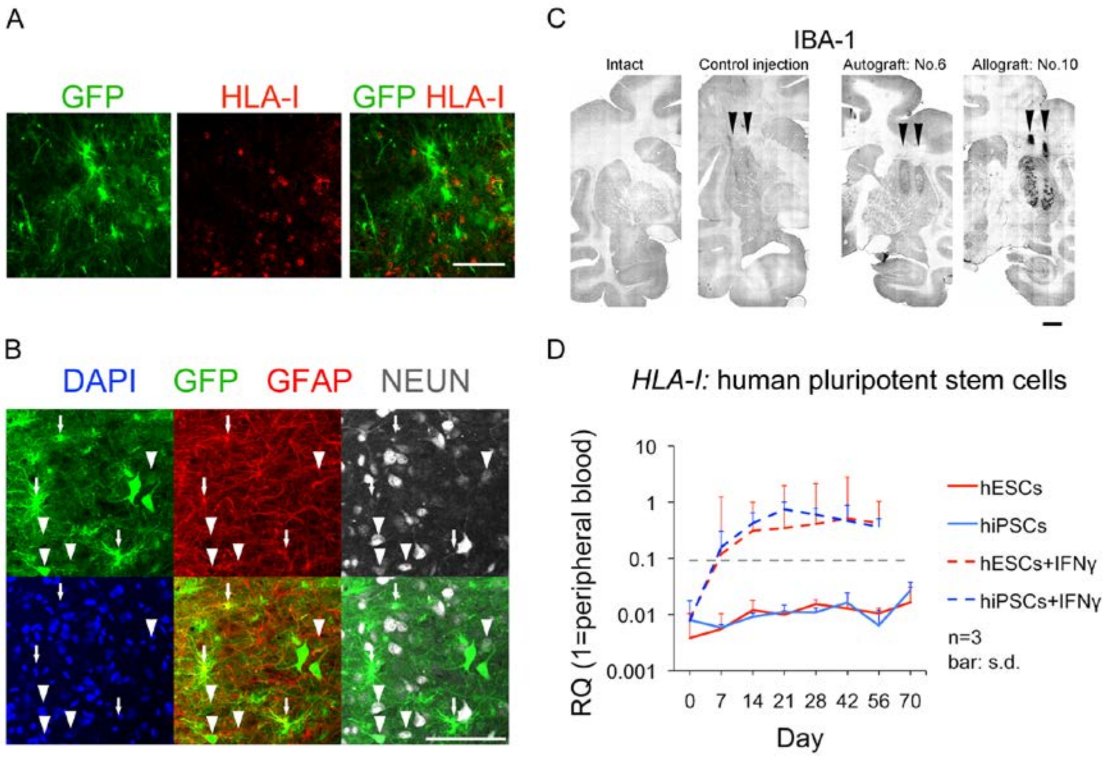

Supplementary Figure Legends

Figure S1: Characterization of iPSCs, related to Figure 1.

Characterization of iPSCs by immunocytochemistry (**A**, **B**, **E**), RT-PCR (**C**, **D**) and genomic PCR (**F**). RT-PCR for Retroviral-iPSCs (from donor No.1 and No.4) showed incomplete silencing of transgenes (**C**, **D**). We selected one clone with the least transgene expression from each animal; 201F for donor No.1 (**A**, **C**; arrowheads, **B**) and T7 (**D**; arrowhead) for donor No.4. Genomic PCR for Episomal-iPSCs (from donor

No.6 and No.8) showed no genomic integration of transgenes (**F**). We selected 969A2 for donor No.6 and 971A1 for donor No.8 (**E**, arrowheads). Scale bars: 200 μm (**A, E**), 50 μm (**B**).

Figure S2: PK11195 PET study, related to Figure 3B.

[^{11}C]PK11195 PET study of all monkeys at one week and one to three months post-surgery. Animal No. 10 (allograft) at three months showed an increase of uptake in the graft area.

Figure S3: Quantitative analyses of IBA1+ and CD45+ cells, related to Figure 3.

Quantitative analyses of IBA1$^+$ (**A**; n=9 tracts and 12 tracts for retroviral and episomal vector, respectively) and CD45$^+$ cells (**B**; n=10 tracts and 12 tracts for retroviral and episomal vector, respectively) in the autografts. Incomplete silencing of transgenes might have caused the immune response associated with higher numbers of Iba1$^+$ and CD45$^+$ cells after transplantation of iPSCs generated with retroviral vectors (****P < 0.0001, by unpaired *t*-test, error bars: s.d.). Variables of cell densities were analyzed

by a two-way ANOVA with factors of auto-allografts and vectors. Again iPSCs established with retroviral vectors caused higher numbers of IBA1+ and CD45+ cells after transplantation ($F(1, 32)=21.06$, $P<0.0001$ (**C**) and $F(1, 36)=4.240$, $P=0.0468$ (**D**) There were no significant interaction effects between vectors and the factor of auto-allograft on the counts of IBA1^{+} cells (**C**; $F(1,32)=3.735$, $p=0.0622$) and CD45^{+} cells (**D**; $F(1,36)=2.271$, $p=0.1406$). n=9 tracts (**C**), n=10 tracts (**D**), error bar: s.d.

Figure S4: Histological analyses and HLA-1 expression of human pluripotent stem cells, related to Figures 2-4.

(**A**) The expression of HLA-1 (MHC-1) was not detected in the GFP^{+} grafted cells *in vivo* (animal No.10). (**B**) The grafted cells (GFP^{+}) differentiated into both astrocytes (GFAP^{+}: arrows) and neurons (NEUN^{+}: arrowheads). (**C**) Low magnified views of the IBA-1-DAB staining. (**D**) *In vitro* analyses of qPCR detected only low level of *HLA-I* during neural cell differentiation from human pluripotent stem cells (embryonic stem cells; ESCs: KhES1, 3, 5 and iPSCs; 836B1, 836B3, 404C2). The dotted line indicates

the expression level of *HLA-I* by HDFa1388; a human dermal fibroblast cell line that was the origin of the iPSCs tested. n=3 independent experiments, error bars: s.d. Scale bars: 100 μm (**A, B**) and 2mm (**C**).

Supplementary Table 1

The cynomolgus macaque *MHC* (*Mafa*) *class I* allele sequences detected by next generation sequencing

Animal number	Simplified allele number	The best matched *Mafa* allele name (IPD accession number)*	Similarity at the nucleotide level	Sequence read number (ratio)
No.1	1-1	Mafa-A1*022:06 (MHC03845)	100%	463 (27.6%)
	1-2	Mafa-B*090:01 (MHC02650)	100%	424 (25.3%)
	1-3	Mafa-B*011:02 (MHC03781)	100%	251 (15.0%)
	1-4	**Mamu-E*02:07 (MHC02638)**	100%	224 (13.4%)
	1-5	Mafa-B*085:01 (MHC02672)	100%	93 (5.6%)
	1-6	**Mafa-B*144:04 (MHC03338)**	99%	81 (4.8%)
	1-7	**Mamu-A4*14:03:01 (MHC01226) / Mamu-A4*14:09 (MHC02186)**	100%	68 (4.1%)
	1-8	Mafa-B*044:01:01 (MHC02731) / Mafa-B*044:03 (MHC02734)	100%	47 (2.8%)
	1-9	Mafa-I*01:14 (MHC02156) etc.	100%	24 (1.4%)
No. 2	2-1	Mafa-B*077:01 (MHC02756)	100%	582 (24.2%)
	2-2	Mafa-B*007:05 (MHC03878)	100%	423 (17.6%)
	2-3	**Mamu-A1*022:01 (MHC01229)**	97%	387 (16.1%)
	2-4	**Mamu-B*013:01 (MHC02244)**	100%	316 (13.1%)
	2-5	Mafa-A1*063:01 (MHC01769) / Mafa-A1*063:02 8MHC01728) / Mafa-A1*063:03:01 (MHC01727)	100%	219 (9.1%)
	2-6	Mafa-B*082:01:01 (MHC03662)	100%	133 (5.5%)
	2-7	**Mamu-E*02:14 (MHC01986)**	100%	112 (4.7%)
	2-8	**Mamu-B*070:02 (MHC02318)**	100%	108 (4.5%)
	2-9	Mafa-B*044:01:01 (MHC02731) / Mafa-B*044:03 (MHC02734)	100%	59 (2.5%)
	2-10	**Mane-B*115:01 (MHC02835)**	100%	41 (1.7%)
	2-11	Mafa-I*01:14 (MHC02156) etc.	100%	24 (1.0%)
No. 3	3-1	**Mamu-B*023:01 (MHC02257)**	99%	731 (32.2%)
	3-2	**Mane-B*089:01 8MHC02837)**	100%	473 (20.8%)
	3-3	**Mamu-A1*032:03 (MHC02807)**	99%	404 (17.8%)
	3-4	Mafa-A1*003:02 (MHC01751)	100%	195 (8.6%)
	3-5	**Mamu-E*02:10 (MHC01629) / Mamu-E*02:11 (MHC01630)**	100%	189 (8.3%)
	3-6	**Mamu-A2*24:01 (MHC01886)**	100%	156 (6.9%)
	3-7	**Mafa-B*030:01:01 (MHC02681)**	99%	62 (2.7%)
	3-8	**Mamu-B*067:02 (MHC02310)**	100%	60 (2.7%)
No. 4	4-1	Mafa-B*018:01:01 (MHC02686) / Mafa-B*018:01:02 (MHC02649)	100%	696 (25.2%)
	4-2	Mafa-B*013:08 (MHC02752)	100%	595 (21.6%)
	4-3	Mafa-A1*045:01 (MHC01716)	100%	362 (13.1%)
	4-4	Mafa-B*017:02 (MHC03998)	100%	318 (11.5%)
	4-5	Mamu-B*007:03 (MHC01237)	100%	226 (8.2%)
	4-6	Mafa-A1*041:01 (MHC01744)	100%	206 (7.5%)
	4-7	Mafa-B*137:03 (MHC01654)	100%	136 (4.9%)
	4-8	**Mamu-B*061:05 (MHC04031)**	98%	107 (3.9%)
	4-9	**Mamu-E*02:14 (MHC01986)**	99%	80 (2.9%)
	4-10	Mafa-A4*01:04 (MHC02021)	100%	35 (1.2%)
No. 6	6-1	**Mamu-A1*019:07 (MHC02800)**	98%	1704 (39.5%)
	6-2	Mafa-B*008:01 (MHC03999)	100%	1299 (30.1%)
	6-3	Mafa-A2*05:42 (MHC03313)	100%	524 (12.2%)

	6-4	Mafa-B*044:01 (MHC02731)	100%	294 (6.8%)
	6-5	Mafa-B*085:01 (MHC02672)	100%	168 (3.9%)
	6-6	Mafa-B*079:02 (MHC03761)	100%	101 (2.3%)
	6-7	Mafa-B*030:08N (MHC02966)	100%	88 (2.0%)
	6-8	Mafa-A2*05:09 (MHC01787)	100%	72 (1.7%)
	6-9	**Mamu-B*062:01 (MHC02304)**	100%	60 (1.4%)
No. 8	8-1	Mafa-B*056:01 (MHC02679)	100%	2145 (30.1%)
	8-2	Mafa-A1*022:06 (MHC03845)	100%	2031 (29.3%)
	8-3	Mafa-B*161:02 (MHC04341)	100%	1395 (20.1%)
	8-4	**Mafa-B*144:04 (MHC03338)**	98%	606 (8.7%)
	8-5	Mafa-B*081:01 (MHC03650)	100%	502 (7.2%)
	8-6	**Mafa-B*079:02 (MHC03761)**	99%	165 (2.4%)
	8-7	Mafa-A2*05:25 (MHC01270)	100%	46 (0.7%)
	8-8	**Mafa-B*088:02 (MHC03760)**	99%	38 (0.5%)
No. 9	9-1	Mafa-B*039:01 (MHC03632)	100%	2991 (40.7%)
	9-2	**Mafa-A1*008:01 (MHC01732)**	98%	1575 (21.4%)
	9-3	Mafa-A1*043:01 (MHC01746)	100%	1563 (21.3%)
	9-4	**Mafa-B*074:01N (MHC02684)**	99%	638 (8.7%)
	9-5	**Mafa-B*007:05 (MHC03878)**	97%	148 (2.0%)
	9-6	Mafa-B*060:04 (MHC03738)	100%	104 (1.4%)
	9-7	**Mafa-B*079:02 (MHC03761)**	99%	90 (1.2%)
	9-8	**Mafa-B*115:02 (MHC04338)**	98%	57 (0.8%)
	9-9	Mafa-B*030:08N (MHC02966)	100%	54 (0.7%)
	9-10	Mafa-B*074:02 (MHC03740)	100%	48 (0.7%)
	9-11	Mafa-B*088:02 (MHC03760)	100%	41 (0.6%)
	9-12	Mafa-A4*01:08 (MHC03326)	100%	39 (0.5%)
No. 10	10-1	**Mafa-A1*018:05 (MHC02593)**	98%	1179 (28.8%)
	10-2	**Mafa-B*041:01 (MHC02031)**	98%	755 (18.4%)
	10-3	**Mafa-B*044:05 (MHC04316)**	99%	610 (14.9%)
	10-4	Mafa-B*083:02 (MHC03754)	100%	573 (14.0%)
	10-5	Mafa-B*082:01 (MHC03662)	100%	352 (8.6%)
	10-6	Mafa-B*069:04 (MHC03877)	100%	304 (7.4%)
	10-7	Mafa-B*030:08N (MHC02966)	100%	201 (4.9%)
	10-8	**Mafa-B*092:01 (MHC02930)**	98%	64 (1.6%)
	10-9	**Mafa-B*060:03 (MHC03380)**	99%	55 (1.3%)

Note; *Bold letters indicate newly discovered cynomolgus macaque MHC (*Mafa*) class I alleles.

Supplementary Table 2

List of primers used for RT-PCR, quantitative RT-PCR, and genomic PCR

Gene Name	Forward (5'-to-3')	Reverse (5'-to-3')	Annealing temperature
**for qPCR**			
MHC-class I (monkey)	TCGTGCGGTTYGAYAGCGACG	CCAGCAYCTCAGGGTGGCCTC	60
HLA-class I (human)	RCTACAAYCAGAGCGAGG	CATCTCCGCAGGGTAGAAG	60
MAP2	GGATCAACGGAGAGCTGAC	TCAGGACTGCTACAGCCTCA	60
CORIN	CACAGCCAGGGTCTGGTGGAATGCAG	GAGAGCTACCACCACATGAATCAAGG	60
TH	TCATCACCTGGTCACCAAGTT	GGTCGCCGTGCCTGTACT	60
GAPDH	GAAGGTGAAGGTCGGAGTC	CATTGATGGCAACAATATCC	60
OCT4	GAGAACAATGAGAACCTTCAGGAGA	TTCTGGCGCCGGTTACAGAACCA	58
**for RT-PCR**			
trans OCT4	GCTCTCCCATGCATTCAAACTGA	CTTACGCGAAATACGGGCAGACA	60
trans SOX2	TTCACATGTCCCAGCACTACCAGA	GACATGGCCTGCCCGGTTATTATT	60
trans KLF4	CCACCTCGCCTTACACATGAAGA	ATACATCCTGTCCGTCCAAGCAGA	60
**for genomic PCR**			
OCT4	GGCTTCTGGCGTGTGAC	TTAGCCAGGTCCGAGGATCA	64
SOX2 and KLF4[a]	CTTGACGCAGTGTCTTCTCCCT	TTCACATGTCCCAGCACTACCAGA	64
L-MYC and LIN28[a]	CTTCCTGGAGCGCAAG	GCGCACGTTGAACCACTTA	64
EBNA1	GGCTTCTGGCGTGTGAC	CTCCTGTTCCACCGTGGGTC	64
OriP	TTCCACGAGGGTAGTGAACC	TCGGGGGTGTTAGAGACAAC	64
NANOG(endo)[b]	TCTCTCCTCTTCCTTCCTCCATG	CTGTTTGTAGCTGAGGTTCAGGATG	64

Note; a, primers were designed to detect the boundary of two genes. b, primers for an endogenous locus were used as loading control.

Supplementary Table 3

List of primary antibodies

Antibodies	Source	Dilution or concentration
For immunocytochemistry and immunohistochemistry		
CD3	Abcam, ab5690	200
CD45	Dako, M0701	200
CD8	Serotec,MCA609G	200
DAT	Millipore, MAB369	1000
FOXA2	SantaCruz, SC6554	500
GABA	SIGMA, A0310	1000
GFP	MBL, M048	200
HLA-ABC (MHC-class Ⅰ)	Hokudo, Ab-46	200
HLA-DP, DQ, DR (MHC-class Ⅱ)	Dako, M0775	500
IBA-1	Wako, 019-19741	400
KI67	Novo CASTRA,NCL-Ki67p	1000
LMX1A	gift from KAN Research Institute	500
NANOG	R&D, AF1997	500ng/mL
NEUN/RBFOX3	Millipore, MAB377	200
NURR1	gift from KAN Research Institute	1000
OCT4	SantaCruz, SC5279	500
PITX3	Millipore, AB5722	200
SEROTONIN	Millipore, MAB352	500
SSEA-4	Millipore, MAB4304	50
TBR1	Millipore, AB10554	2000
TH	Millipore, AB152/MAB318	400
TRA-1-60	Millipore, MAB4360	100
TUJ1/βTUBULIN CLASSⅢ	Covance, MMS-435P	1000
For flow cytometry		
βTUBULIN CLASSⅢ	BD, 560394	20
HLA-ABC	BD, 560169	5
PSA-NCAM	Millipore, MAB5324	100
OCT4	BD, 560253	5
